# Evaluating AI Versus Examiner Feedback in Ophthalmology Exit Examinations: A Pilot Study

**DOI:** 10.7759/cureus.87591

**Published:** 2025-07-09

**Authors:** Binita Panchasara, Andrew Malem, Paul Calcraft, Sean Zhou

**Affiliations:** 1 Ophthalmology, North West Anglia Foundation Trust, Peterborough, GBR; 2 Paediatric Ophthalmology, Cleveland Clinic, Abu Dhabi, ARE; 3 Software Development, iPrepBuddy, London, GBR; 4 Ophthalmology, Norfolk and Norwich University Hospital, Norwich, GBR

**Keywords:** chat gpt, clinical skills education, deep learning artificial intelligence, gpt-3.5, gpt-4, health communication, large language model(llm), ophthalmology, oral examination, teaching feedback

## Abstract

Objectives

This pilot study aims to compare the quality of feedback provided by generative artificial intelligence (AI) and an official Royal College Examiner for simulated clinical and communication scenarios designed to prepare candidates for the Royal College of Ophthalmologists Part 2 Oral Examination.

Design

Utilising GPT 3.5 and 4 (OpenAI, San Francisco, CA, USA), an interactive web-based platform has been created that is able to simulate both patient and examiner roles in oral examination scenarios and simultaneously provide feedback on a candidate’s performance. Feedback was provided solely using GPT-4 in combination with prompt techniques. A standardised patient was used to enact five clinical and communication scenarios that were each assessed by both the AI and a Royal College of Ophthalmologists Examiner. The transcripts from these sessions were thematically analysed using NVivo software (Lumivero, Burlington, MA, USA) to compare the quality and content of the feedback from both sources.

Main outcome measures

To determine the similarities and differences in the content and structure of feedback provided by AI (Examiner A) and a Royal College Examiner (Examiner B) in the context of preparing candidates for the Fellowship of the Royal College of Ophthalmologists (FRCOphth) Part 2 Oral Examination.

Results

The results reveal that while both Examiner A and Examiner B provide feedback on similar themes, such as empathy, communication clarity and systematic clinical reasoning, their approaches differ. Examiner A’s feedback was more structured and often referenced specific frameworks, offering detailed, protocol-driven guidance. In contrast, Examiner B’s feedback was more practical and context-specific, focusing on real-world applications and providing nuanced insights shaped by experiential knowledge.

Conclusion

The findings suggest that generative AI has the potential to complement traditional oral exam preparation methods by providing easily accessible, structured and scalable feedback which could provide an early foundation of learning for candidates. It may be particularly useful for those unfamiliar with the specific requirements of Royal College examinations.

## Introduction

At the heart of healthcare is a need for continuous learning and training, which is constantly being adapted to meet the rapidly changing landscape of evidence-based practice and social requirements. Several large language models (LLMs) have been tested to different degrees in healthcare, with models such as GPT-4 (OpenAI, San Francisco, CA, USA) and PaLM2 (Google, Inc., Mountain View, CA, USA) showing real promise in the field of postgraduate education [1-5].

As a general-purpose model, GPT-4 has demonstrated advanced reasoning and content generation abilities that rival those of experts in fields such as ophthalmology, internal medicine and emergency medicine [6-8]. Its accessibility enables even non-experts to generate tailored learning content and simulations for exam preparation.

Post-graduate medical examinations are notoriously demanding, requiring substantial time, effort and financial investment from already overburdened doctors. Oral examinations such as OSCEs, VIVAs and PACES, which typically represent the final stage of the exam process, can be particularly challenging to prepare for. Candidates often need to find peers to practise with or enrol onto expensive courses to gain the necessary practice and build their confidence. While studies have explored the capability of LLMs like GPT-4 to answer post-graduate level exam questions, there is currently no data on the use of such systems to provide interactive practice and feedback of sufficient quality to prepare individuals for their oral examinations. Such a model would provide a flexible and easily accessible means by which candidates could practice their communication skills individually, in order to gain the knowledge and confidence necessary to tackle in-person sessions or indeed the exam itself. Such a system may additionally have the potential to help international medical graduates integrate more easily into the UK healthcare system.

Early small-scale studies have demonstrated the ability of GPT models to help clinicians develop their communication skills to good effect in challenging scenarios. One study demonstrated promising results when GPT was used to test radiographers' communication skills with claustrophobic patients during an MRI scan [9]. Others have shown the potential to evaluate specialist communication skills in the context of a high-stress encounter or when discussing patient interventions [10]. Despite these advancements, however, concerns persist over the use of LLMs in medicine. Chief among these are the risks of propagating misinformation and the unreliability of outputs, which are the result of both the training datasets and the probabilistic nature of LLMs. Prompt engineering is one approach to reducing such risks by guiding generalist models such as GPT-4 to generate responses that are more accurate, reliable and clinically appropriate [1].

This paper looks at a conversational AI platform designed to enhance communication and clinical reasoning skills, using the Royal College of Ophthalmologists Part 2 Oral Examination as a benchmark. The primary objective is to evaluate and compare the feedback provided by AI in interactive clinical and communication scenarios against the feedback given by an official Royal College of Ophthalmologists Examiner. Through this pilot study, we aim to assess the quality of the AI’s feedback and better understand the role that generative AI can have in oral exam preparation.

## Materials and methods

A platform was built combining existing generative LLMs (GPT 3.5 and 4) with computer voice recognition and synthesis to simulate oral examination scenarios for the Part 2 Fellowship of the Royal College of Ophthalmologists (FRCOphth) examination. In each communication scenario, the AI simulates both the patient and examiner, providing personalised feedback to the candidate at the end of the interaction. The clinical scenarios involve the AI acting as the examiner alone, asking questions on a specific topic, while simultaneously analysing the candidate’s performance, which is similarly fed back to them at the end. The feedback component is modelled using GPT-4 in combination with consistent and carefully structured prompting, which utilised techniques including series, parallel, few-shot and generated knowledge prompting in addition to retrieval augmented generation (RAG).

A standardised patient was used to simulate five clinical and communication scenarios which were recorded and evaluated by a Royal College Examiner (Examiner B) as well as simultaneously by the AI. Examiner A was blinded to the feedback outcomes of Examiner B and vice versa.

The transcript was thematically categorised, and qualitative content analysis was carried out utilising NVivo software (Lumivero, Burlington, MA, USA). Analysis of the final defined themes was examined between the LLM-derived and examiner-derived feedback transcripts.

## Results

To summarise the findings of our thematic analysis, both examiner A (AI-powered) and examiner B provided feedback on similar themes, but may have had different approaches to this. Below is a table (Table 1) outlining the comparisons of their performance on the communication and clinical scenarios.

**Table 1:** Table comparing the performance of Examiner A and Examiner B for the clinical and communication scenarios.

	Similarities	Differences
Communication	Both emphasise empathy and professionalism in patient interactions. They agree on the importance of clarity in communication, the need to involve patients, and comprehensive history-taking. Both value structured communication, though Examiner A explicitly recommends frameworks like SPIKES. They stress the need for clear follow-up planning.	Examiner A offers more detailed and structured feedback, often rooted in specific frameworks. Examiner B gives more practical, situation-specific advice, focusing on how to improve in real-world scenarios. For example, while both discuss breaking bad news, Examiner A suggests using structured protocols, whereas Examiner B advises on situational methods.
Clinical	Both emphasise a systematic approach in clinical reasoning and image interpretation. They stress the importance of detailed descriptions and effective categorization of clinical information. They highlight the necessity of sensitivity and clarity in communication with patients/parents. Both recommend further study to enhance clinical skills and acknowledge the role of guidelines and landmark trials.	Examiner A focuses on broader and more systematic categorizations, while Examiner B provides more specific and practical guidance. Examiner B is more precise in advising on details for image interpretation and suggests specific tests during clinical examinations. Examiner A often recommends further study, whereas Examiner B concentrates on refining specific knowledge areas.

Summary of results

While both examiners provide valuable feedback, Examiner A (AI) is more structured and often protocol-driven, offering detailed insights based on specific frameworks, while Examiner B (Human) focused more on the specific situation. No hallucinations were noted in the AI feedback responses. Please see attached Appendix 1 for the full results of qualitative content analysis.

## Discussion

This pilot study aimed to evaluate the quality of feedback provided by a conversational AI model compared to that of a Royal College Examiner in the context of preparing candidates for the Royal College of Ophthalmologists Part 2 Oral Examination. Our findings highlight that both the AI and human examiner provided feedback on similar themes both in the communication and clinical scenarios, although their approaches differed. The AI provided more structured, framework-driven feedback whereas the human examiner offered more practical, context-specific advice. While the AI demonstrated potential for consistent and standardised feedback, the human examiner provided more nuanced insights tailored to real-world applications. This reflects the inherent differences in their respective interactions and cognitive pathways, with the AI’s feedback being driven by pattern recognition and reference to specific written frameworks, model answers and guidelines, while the human examiner’s insights are shaped by experiential knowledge, empathy and a deeper understanding of context and real-world complexities.

A key strength of this preliminary study is the direct comparison between AI-generated feedback and that provided by an official examiner, which is an important first step in understanding the potential role that AI can have in supporting doctors preparing for oral medical board examinations. With the rapid advancement of AI and LLMs, conversational AI is becoming an area of growing interest in medical education. The findings presented here contribute to the emerging body of evidence needed to critically assess and validate its use. While our results demonstrate GPT-4’s ability to produce relevant, detailed and structured feedback with careful prompting, it remains important to contextualise its performance alongside other AI models and platforms. A meaningful comparison is nonetheless difficult, as most existing studies focus on the use of LLMs to answer medical exam questions rather than their ability to generate feedback in simulated clinical interactions.

GPT-3, the predecessor of GPT-4, has been widely studied for its natural language generation capabilities; however, its outputs were often less accurate in domain-specific contexts due to limited reasoning depth and factual consistency. GPT-4 alternatively has been shown to be superior in both coherence and factual grounding, particularly when prompted effectively, as demonstrated by our results [7,11]. At the time of our study being conducted, GPT-4 was the most practical model to integrate into the test scenarios. While other LLMs such as Gemini/Bard and earlier versions of GPT were considered, GPT-4 provided the optimal balance between output quality and implementation feasibility.

Of particular interest is the prospect of using domain-specific models to improve the accuracy and consistency of AI-assisted medical education learning tools. Google’s Med-PaLM2 is one such model that demonstrated a high degree of accuracy, rivaling even that of experts, in answering USMLE-style questions [8]. More recently, the development of open-source models such as OpenBioLLM-8B and Palmyra-Med, have been preliminarily shown to out-perform existing leading models including Med-PaLM2 and GPT-4 in answering medical exam questions [7,8,12-15]. While these prospects are indeed exciting, there remains a lack of high-quality evidence to support their safe and effective use in healthcare, particularly in the context of interactive learning.

The study does have its limitations, the first of which is the small sample size and limited range of scenarios which restrict the generalisability of the findings. The study’s reliance on a single AI model and a single human examiner additionally limits the generalisability of findings, as it does not account for the variability in feedback styles and effectiveness across different models and examiners, and introduces a potential source of bias. Furthermore, the study did not assess the long-term impact of the AI's feedback on exam performance or clinical practice, leaving questions about its value in real-world settings.

One significant limitation to using general LLMs is their inherent variability and risk of hallucinations. To address this, we used a more powerful model (GPT-4) for feedback generation and applied consistent and carefully structured prompts, using a combination of techniques, across all scenarios. The prompts provided structured answer frameworks and guidelines, minimising reliance on the model’s general pretraining and reducing the extent to which clinical reasoning was required. While minor variation in phrasing was observed, the core content, thematic accuracy and feedback quality remained stable. Though this may be reasonable for the purpose of this small study, further research is necessary to quantify output variability more systematically for a wider context of clinical scenarios. In the meantime, users of AI-based learning tools should be aware of the potential for misinformation and corroborate content with trusted clinical sources.

A significant difference between the findings of this study and others is the depth of feedback provided by the AI model. While previous reports suggest that AI can provide broad feedback on performance, this study demonstrates that AI can deliver more objective, structured and systematic guidance based on established clinical frameworks [9,10]. Taking this into consideration, a hybrid model of learning may offer the greatest benefit whereby AI-based educational tools are used to complement traditional learning methods to address gaps in the AI's ability to provide “real-world” training and to help reduce the risk of promoting mechanical or overly formulaic responses should one become overly reliant on AI-generated content.

The use of an AI-based interactive educational platform for clinical exam preparation may be beneficial in helping doctors to build a foundational understanding of communication and clinical reasoning frameworks before engaging in traditional in-person simulation, which may be of particular benefit for international medical graduates or those unfamiliar with the specific expectations of Royal College examinations. For clinicians, the use of AI could mean more accessible and flexible learning options, with the potential to reduce the financial and time burdens associated with traditional preparation techniques. Furthermore, AI could help democratise access to high-quality educational resources, making them more available to a broader range of learners, including those in resource-poor settings.

## Conclusions

Overall, this study provides an early insight into the potential role of generative AI in medical education, highlighting its strengths while acknowledging the need for further research and thoughtful integration. This study marks the beginning of a larger initiative to generate robust, quantitative evidence on the effectiveness of AI as a tool for oral examination training. Beyond this, there is a need to explore the long-term effects of AI-based learning on communication skills development, exam performance and ultimately, its impact on clinical practice.

## Appendices

Appendix 1

COMMUNICATION SCENARIOS FEEDBACK

Theme 1: Empathy and Emotional Support to Patient

The two feedback providers demonstrated commendable empathy by listening attentively to patients, acknowledging their concerns, and maintaining professionalism. Examiner A mentioned that medical personnel excelled in showing empathy through calm communication and validating patient fears but could improve by more explicitly addressing and verbalizing emotional distress. Examiner B mentioned that medical personnel effectively balanced empathy with clear communication about necessary restrictions and patient safety, though there was a need for clearer articulation regarding driving limitations and the full implications of medical decisions.

Example quotes:

AI response: “The professionalism and calmness that the doctor demonstrated were commendable. The doctor listened attentively to the patient's responses and demonstrated empathy”

AI response: “The doctor acknowledged the patient's frustration but did not address his need to see in order to drive his wife to her appointments. Offering solutions for this could have lessened the patient's anxiety”.

Human examiner response: “Good empathy shown, but important that doctor is honest with patient about the rules around driving. I would say that temporarily driving has to be halted for the safety of himself and others and the last thing we want him to do is have a car accident or a fine”.

Theme 2: Preparation and Delivery of Bad News to Patient

Together, they both highlighted the necessity of preparing patients before delivering bad news. Examiner A provided feedback on the importance of preparing patients before delivering bad news. The examiner noted that in several scenarios, the doctor failed to assess the patient's current knowledge or readiness to receive serious information, and did not provide a "warning shot" to help the patient mentally prepare. The recommendation was to first gauge the patient’s understanding and preferences about how much information they wish to receive before breaking the bad news. This includes providing a preliminary indication that serious news is forthcoming.

Examiner B similarly emphasized the need for a "warning shot" before delivering bad news. The feedback indicated that delivering the news without preparing the patient could lead to shock or distress. Examiner B suggested introducing the bad news by first indicating that there were concerning findings that needed further investigation or explaining that some changes had been observed which might explain the patient’s symptoms.

Example quotes:

AI response: “They didn't fully prepare the patient for the difficult conversation. The doctor could have asked about the patient's perception, and how much information he would like to know before delivering the bad news”.

Human examiner response: “The doctor breaks the news of bilateral disc swelling. There wasn’t really a warning shot and so I would have explained that we have examined the eyes and we do see some changes that might be related to the headaches and nausea”.

Theme 3: Information Provision and Clarity

Both Examiner A and Examiner B stressed the importance of clear and structured communication in medical consultations. Examiner A noted several areas for improvement in the clarity of explanations provided by the doctor. Specifically, the feedback indicated that the doctor failed to clarify why the patient couldn't drive, did not adequately explain complex medical terms, and did not provide a clear description of necessary tests. The feedback emphasized the need for a structured conversation, including summarizing key points and using signposts to guide the patient. The doctor was also encouraged to simplify explanations of medical conditions and procedures to reduce patient anxiety.

Examiner B similarly highlighted the need for clearer explanations and structured communication. Feedback pointed out that the plan for future steps should have been more clearly outlined, and that complex concepts, such as the implications of high blood pressure and the anatomy of the optic nerve, should be explained in a more understandable manner. Additionally, clear signposting and a structured outline of the next steps were recommended to ensure the patient fully understands the process and future actions.

Both examiners agree that improving the clarity and structure of explanations can help alleviate patient anxiety and ensure better understanding of their medical situation.

Example quote:

AI response: “The explanation of why the patient can't drive was not clear enough. The doctor showed a commitment to help the patient but did not explicitly state so. The conversation needed a bit more structure - summarizing key points and using signposts to guide the patient through the process”.

AI response: “Language was simple and patient-friendly, but complex medical terminologies were not adequately explained”.

Human examiner response: “At the end, I think signposting the future plan could have been clearer, in other words, he wants a clear plan outlined. I would have stated exactly what the next steps are - imaging/blds/neurology and starting some tablets to reduce his CV risk factor”.

Theme 4: Gathering Comprehensive Patient History

Gathering a comprehensive patient history is a fundamental component of effective medical practice. This process involves collecting detailed information about a patient's personal background, medical and family history, current medications and treatments, lifestyle factors, symptoms, and social circumstances. By obtaining a thorough and holistic understanding of these elements, healthcare providers can gain crucial insights into the patient’s overall health and well-being. This information is essential for making informed diagnostic and treatment decisions, tailoring care plans to individual needs, and improving patient outcomes.

Sub-theme 1: Medical History and Risk Factor

Together, the feedback from both examiners underscores the necessity of thorough patient history, including detailed inquiries into neurological issues, risk factors, medication use, and general health conditions.

Examiner A noted several gaps in the patient history inquiry. The doctor failed to ask about critical neurological issues, such as vision problems, speech issues, and mobility struggles, as well as important risk factors like high cholesterol, high blood pressure, diabetes, previous cardiovascular events, and smoking habits. Additionally, the history of optic neuritis, the speed of onset of visual loss, pain on eye movement, and color vision were not addressed. The doctor also missed asking about past medical history, medication history, and recent infections or immunizations, which are essential for an accurate diagnosis.

Examiner B pointed out that essential aspects of patient history, such as past high blood pressure and current medication use, should be explored. They emphasized the importance of asking about other symptoms, potential causes of symptoms, and any relevant neurological history. Specifically, in cases suspecting optic neuritis, inquiring about a history of optic neuritis, weakness, numbness, onset of visual loss, pain on eye movement, color vision, and any recent infections or immunizations is crucial.

Addressing these areas comprehensively can significantly enhance diagnostic accuracy and treatment planning.

Sub-theme 2: Social and Medication History of the Patient

Gathering a comprehensive patient history should include not only medical but also social and medication-related details. Feedback from Examiner A and Examiner B underscores the importance of addressing these aspects.

Examiner A pointed out that the doctor neglected to ask about the patient’s social circumstances beyond acknowledging an upcoming wedding. Key social factors such as the patient’s work, living situation, and driving routine were not explored. Additionally, there was a lack of depth in discussing the patient's social and medication history, which is essential for a thorough understanding of their situation. The interaction was criticized for being poorly structured and missing several important areas, particularly in terms of collecting a complete medical, social, and medication history.

Examiner B did not provide specific feedback on social history in the provided excerpts, but their emphasis on detailed inquiries about symptoms, medication, and general health complements the need for a comprehensive patient history.

Together, the feedback highlights that a thorough patient history should encompass detailed medical, social, and medication-related information. Both examiners stress the need for a well-structured approach to ensure all relevant aspects are covered to inform diagnosis and treatment effectively.

Theme 5: Patient Engagement and Structured Communication

Patient engagement and structured communication are fundamental components of effective healthcare delivery. Engaging patients in their own care involves actively involving them in healthcare decisions, encouraging open dialogue, and ensuring they fully understand their conditions and treatment options. Structured communication, on the other hand, refers to the systematic and organized approach healthcare providers use to gather comprehensive patient information and explain medical conditions and procedures in clear and understandable terms. Together, these practices enhance the quality of care by fostering a patient-centered environment where communication is clear, comprehensive, and conducive to better health outcomes.

Sub-theme 1: Checking Understanding and Exploring Patient Concerns

In the feedback provided by both Examiner A and Examiner B, there is a clear emphasis on the importance of engaging with patients by encouraging them to ask questions and address their concerns.

Examiner A emphasizes the importance of doctors encouraging patients to ask any further questions, particularly in challenging circumstances, as this helps in refining communication skills which are vital in patient care. The feedback mentions that doctors should reassure patients and acknowledge their concerns, such as those related to vision and glaucoma, to ensure they feel heard and understood. The examiner also suggests that when sensitive issues like a family history of serious illness (e.g., the patient’s father's brain cancer) are mentioned, it would be appropriate to explore these further to better understand the patient’s worries about their own health. Examiner A notes the importance of checking the patient’s understanding of the information provided, especially when discussing serious health conditions, and ensuring that any additional questions or concerns are addressed.

Examiner B consistently advises that doctors should end consultations by rechecking the patient’s understanding, asking about any concerns or questions they might have, and ensuring that the patient fully comprehends the next steps. Examiner B highlights the value of asking patients about their pre-existing knowledge of their condition (e.g., asking if they’ve heard of glaucoma) as this can help tailor the explanation to their needs and clarify any misconceptions.

Furthermore, Examiner B recommends encouraging open dialogue by asking the patient if they have any ideas about what might be happening, as this can reveal any pre-existing concerns or information the patient might have, which is important for progressing with the explanation. Emphasizes the need to clearly explain the next steps and to summarize the discussion, ensuring the patient has no remaining questions or concerns and fully understands what has been discussed.

Sub-theme 2: Structured Communication Framework and Follow-up

The examiners stress the significance of structured communication and planning in patient care. Examiner A focuses on the use of the SPIKES protocol to deliver bad news effectively and ensure the patient’s emotional needs are addressed, while Examiner B highlights the importance of outlining a clear plan for managing the patient’s condition and ensuring follow-up care. Together, they emphasize the need for a systematic approach in both delivering difficult information and guiding patients through their treatment journey.

Examiner A frequently emphasizes the importance of using the SPIKES protocol (Setting, Perception, Invitation, Knowledge, Emotions, Summary) for delivering bad news. This structured approach ensures that the patient fully understands the situation and feels supported emotionally. Examiner A also highlights the need for clear communication of follow-up plans, including advising patients on necessary actions like informing the DVLA or refraining from driving. The importance of scheduling follow-up appointments to monitor the patient’s condition and provide ongoing support is also stressed.

Examiner B underscores the importance of outlining clear plans for managing the patient’s condition, particularly when it comes to controlling blood pressure or coordinating with specialists like neurologists. Examiner B also emphasizes the need for clear communication regarding the next steps in treatment and the importance of regular follow-up to monitor progress and ensure proper adherence to the treatment plan.

CLINICAL SCENARIOS FEEDBACK

Theme 1: Detailed and Systematic Image Description

Both parties stress the importance of a systematic and detailed approach when interpreting and describing medical images in a clinical setting.

Examiner A emphasizes the importance of providing a systematic and detailed description when interpreting medical images during exams. They highlight the need to identify specific details, such as describing an optical coherence tomography (OCT) scan as showing cystoid macular oedema or elaborating on a Hess chart by discussing specific muscle actions. Examiner A also suggests the inclusion of additional assessments, such as optic nerve function tests and bedside tests like blood pressure and blood glucose measurements, to ensure a comprehensive evaluation.

Examiner B echoes the need for precision and detail in describing medical images. They suggest specifying the eye being examined and clearly identifying the image type, such as a Hess chart or a color fundus photograph. Examiner B also advises being more explicit about the findings, such as mentioning macula appearance or detailing subretinal and intraretinal fluid in an OCT scan.

Example quotes:

AI response: “When describing an image in an exam setting, it's important to be systematic and detailed. The image of a normal retina should be identified as a colour retinal (or fundus) image of the right eye”.

Human examiner response: “I would be a little more specific when describing what the image demonstrates therefore I would say it is a colour fundus photograph of the right eye. Candidate did mention disc and blood vessels but should also comment separately on macula appearance”.

Theme 2: Comprehensive Differential Diagnosis

Examiner A and Examiner B underscore the need for a structured, detailed, and specific approach when discussing differential diagnoses and describing clinical conditions.

Examiner A emphasizes the importance of providing detailed and structured differential diagnoses in clinical scenarios. They suggest categorizing causes into specific groups, such as iatrogenic, inflammatory, vascular, structural, pharmacological, and genetic, to enhance clarity and thoroughness. Examiner A also advises expanding on the details when describing specific conditions or findings, such as retinal hemorrhages, differentiating chronic versus acute nerve palsy, and categorizing congenital and acquired causes for clarity.

Examiner B complements this approach by recommending the inclusion of specific clinical details and emphasizing the need to start with the most common causes when discussing differential diagnoses. Examiner B suggests a systematic approach to describing clinical signs, such as using established laws (Hering’s and Sherrington’s) to explain muscle innervation and being precise in quantifying conditions like torsion. They also stress the importance of specificity in describing the features of conditions like diabetic retinopathy and macular edema.

Examiner A focuses on the importance of categorization and thorough exploration of possible causes, while Examiner B highlights the value of starting with the most common causes and incorporating precise clinical details. Together, they advocate for a methodical and comprehensive approach to ensure accurate and effective clinical reasoning.

Example quotes:

AI response: “Your differential diagnoses for cystoid macular edema were broadly correct. However, you are required to provide more structured and detailed explanations. For example, you could categorize the causes as iatrogenic (post-surgical or laser-associated), inflammatory (uveitis-associated), vascular (e.g., diabetic maculopathy or due to retinal vein occlusions or choroidal vascular disease), structural (epiretinal membrane or vitreomacular traction), pharmacological (certain eye drops or chemotherapeutics), and genetic causes (like retinitis pigmentosa)”.

AI response: “Your differentiation on chronic versus acute fourth nerve palsy was brief. In such cases, you might want to provide more details such as the features of a chronic fourth nerve palsy: compensatory head tilt away from the affected side, large vertical fusion range (10-20 prism diopters), facial asymmetry, and lack of subjective excyclotorsion”.

Human examiner response: “Candidate mentioned head tilt which may be present in old photos. This is correct although head tilts do occur acutely but evidence of longstanding head tilt since childhood is correct. I would also mention that enlarged vertical fusion ranges develop with longstanding superior oblique palsy (SOP) and eventually there may be a spread of comitance”.

Theme 3: Structured Examination Approach and Documentation

The examiners advocate for a detailed, systematic, and thorough approach to clinical examinations and documentation. Examiner A focuses on the broader importance of structured assessments, risk categorization, and the use of proformas, while Examiner B provides specific recommendations on how to conduct and document these examinations with precision. Together, they emphasize the need for meticulous attention to detail and the application of standardized practices to ensure comprehensive patient care and accurate clinical documentation.

Examiner A emphasizes the importance of conducting thorough and systematic examinations, particularly in high-stakes scenarios. They stress the need to consider a comprehensive range of risks and potential complications, both intra-operative and post-operative. Examiner A also highlights the value of reviewing landmark trials to inform medical practice and recommends the use of specific proformas (e.g., Royal College of Ophthalmologist Proforma for Non-Accidental Injury) to ensure all relevant details are documented. Additionally, they advise adopting a more structured approach to answering questions and preparing for exams by systematically covering all key details.

Examiner B builds on this by providing specific examples of what should be included in clinical examinations and documentation. They recommend being precise when describing examinations of optic nerve function, such as specifying checks for visual acuity, color vision, and visual fields. Examiner B also suggests a detailed approach to documenting findings, including the presence of retinal hemorrhages, pupil dilation, and the time of the exam. They emphasize the importance of using established guidelines and templates to ensure that all relevant information is accurately recorded.

Theme 4: Management and Treatment Plans

Both Examiner A and Examiner B advocate for a detailed and nuanced approach to the management and diagnosis of ocular conditions. Examiner A highlights the need for a broad range of treatment options and thorough screening, while Examiner B focuses on specific diagnostic and management strategies, emphasizing the importance of personalized care and urgent intervention when necessary.

Examiner A emphasizes the importance of considering a wide range of treatment options for managing conditions like cystoid macular edema and fourth nerve palsy. They suggest not only using anti-inflammatory eye drops but also considering other medical and surgical interventions based on the specific cause of the condition. Additionally, they highlight the need for a comprehensive approach to screening programs and stress the importance of promptly addressing abnormal imaging findings. Examiner A also advises adding depth and structure to management strategies and descriptions.

Examiner B provides more specific guidance on management and diagnostic approaches. They recommend tailoring treatment based on symptoms and signs, such as using prisms for minor deviations and considering surgery for significant issues. Examiner B emphasizes the importance of ruling out serious conditions like AION or GCA, conducting urgent tests and imaging, and addressing any concerns about potential risks and side effects of treatments like Ozurdex.

Together, they underscore the importance of a comprehensive and structured approach to both treatment and diagnosis, with attention to detailed risk management and timely action.

Theme 5: Communication with Patient and Parent

The two examiners agree that handling conversations with parents about further testing should be done with sensitivity and clarity. Examiner A highlights the need for careful communication with reassurances and empathy, while Examiner B supports a straightforward approach, focusing on the necessity of observation and investigation without overwhelming parents with specifics.

Examiner A commends the sensitive and appropriate manner in which the conversation about further testing was handled with the parents. They emphasize the importance of communicating next steps with reassurance and empathy, refining how these are conveyed to ensure clarity and support.

Examiner B agrees that a reasonable approach is to avoid excessive detail with parents but to clearly state that a period of observation and investigation is required.

In conclusion, examiners A and B emphasize the importance of balancing sensitivity with clear, supportive communication in such discussions.

Example quotes:

AI response: “Your approach in telling parents about the need for further testing was indeed a sensitive and appropriate one. This is a very difficult conversation to have and it's important that it be done in a careful and considerate manner.”

Human examiner response: “Answer reasonable, not going into specifics with parents but saying need period of observation and investigation is appropriate”.
